# Including mental health care in a model of European health system

**DOI:** 10.1017/S2045796023000057

**Published:** 2023-02-20

**Authors:** Livio Garattini, Angelo Barbato, Barbara D'Avanzo, Alessandro Nobili

**Affiliations:** Department of Health Policy, Institute for Pharmacological Research Mario Negri IRCCS, 20156 Milan, Italy

**Keywords:** Health economics, mental health, quality of care, social and political issues

## Abstract

The management of a health system is a matter of economics and business administration because of the costs induced by goods and services delivered. Economics teaches us that the positive effects induced by competition in free markets cannot be expected in health care, which is a classic example of market failure from both demand and supply sides. The most sensible key concepts to refer for managing a health system are funding and provision. While the logical solution for the first variable is universal coverage through general taxation, the second one requires a deeper understanding. Integrated care is the modern approach that better supports the choice in favour of the public sector also for service provision. A major threat against this approach is dual practice legally allowed for health professionals, which inevitably raises financial conflicts of interest. An exclusive contract of employment for civil servants should be the sine qua non for providing public services effectively and efficiently. Integrated care is particularly important for long-term chronic illnesses associated with high levels of disability, such as neurodegenerative diseases and mental disorders, where the mix of health and social services needed can be very complex. Nowadays the growing number of community-dwelling patients with multiple physical and mental health needs is the major challenge for the European health systems. This happens also in public health systems, which should provide universal health coverage in principle, and the case of mental disorders is striking. In the light of this theoretical exercise, we strongly believe that a public National Health and Social Service should be the most indicated model for both funding and providing health and social care in modern societies. The big challenge of the common model of European health system here envisaged would be to limit the negative influences of politics and bureaucracy.

## Introduction

The health system is a key frame in any developed nation. Tightly related in all settings to health care professions, starting from physicians and nurses, the management of a health system is also a matter of economics and business administration because of the costs induced by goods and services delivered. As a consequence, health care has always been a debated subject in politics, often open to misleading ideologies and demagogies.

Here, we try to put order in the debate on health from the policy point of view (Saltman, 2018; Tynkkynen and Vrangbæk, 2018). We will consider mental health care as a case in point to address the challenges related to the emerging needs raised by the social determinants of health, the long-term care and the chronic care model. The final goal of our effort is to support the proposal for a common model of European health system, based on a few but solid theoretical principles.

## The myth of competition in health

Economics teaches us that the positive effects induced by competition in free markets cannot be expected in health care, by definition (Garattini and Padula, 2019). In fact, health care is a classic example of market failure from both demand and supply sides, the two key concepts of economics.

From the demand side, health care users cannot be considered the common consumers described in economics, who shop around for buying the best product at the lowest cost (Garattini and Padula, 2018*a*). Not being fully informed in health care by default, a patient fills the information gap by devolving to a physician the decision on what goods/services to use. Moreover, the patient can neither be considered a rational consumer, since the (real or perceived) illness makes them weak and vulnerable, hence often prone to financial blackmailing by health care providers.

From the supply side, real competition requires a reasonable number of providers offering the same products/services and operating in similar conditions (Barros *et al*., 2016). Beyond the general evidence that these necessary conditions are nowadays scant in many markets, the unlikely presence of them in health care might even reflect an irrational situation at local level, e.g. some similar hospitals located in the same low-populated area.

In the light of these insurmountable barriers against competition, the logical consequence is that prices cannot come from naturally matching demand and supply in health care. Even though prices are fixed ex ante through tariffs, as it happens in many European countries for hospital admissions – the so-called DRG tariffs imported by the US system (Thompson, 1983) – setting prices artificially is necessarily an arbitrary exercise, which eventually leads to financial distortions and irrational allocation of resources among health care players (Siciliani *et al*., 2017). Furthermore, fee-for-service systems dramatically increase administrative costs, entailing a systematic auditing on how health care providers use them and a periodic updating of the tariffs values (Garattini and Padula, 2019).

## The realistic approach of funding and provision

Once market competition is ruled out, the most reasonable key concepts to refer for managing a health system are funding and provision. While the logical solution for the first variable is quite easy to find out, the second one requires a deeper understanding.

The most rational criterion to apply for funding a health system at macro level is universal coverage through general taxation. In principle, the State is the best ‘insurer’ to cover the ‘illness risk’ of its citizens, being able to spread the risk on the whole population regardless of the tax system adopted.

The concept of expenditure obviously deals with the cost of delivering health care services, with ‘providers’ that are conventionally a mix of public and private bodies in the Western European countries. At the micro level, the discipline to refer for managing health care organisations is business administration, especially planning and budgeting. The term ‘company’, usually associated to the private sector in common languages, can be applied to any kind of employer, including public administration, with the aim of enhancing efficiency in labour organisation such as in a private company.

While it is pretty clear to opt for a public health system for financing health care, the choice between public and private actors for providing health care is less straightforward. In principle, a private company must make profit or cover costs at worst. Therefore, it is not surprising if, for example, private hospitals usually focus on the most profitable and/or least costly treatments (Tynkkynen and Vrangbæk, 2018). On the other hand, it is fair to recognise that public organisations are often open to strong political pressure in making their decisions and slowed down in their administrative procedures by the rigid bureaucracy that traditionally characterises the public sector (Saltman, 2018).

## The goal of integrated care and the scare of dual practice

Integrated care (IC) is the modern approach that better supports the choice in favour of the public sector also for service delivery (Garattini *et al*., 2022*a*). IC is a concept of common sense that emerged in the literature at the beginning of the new millennium and has undoubtedly laudable aims for people. Striving for combining parts to form a whole, IC implies a full collaboration among the professionals involved in modern health and social services to struggle against the widespread fragmentation of services delivered. A major threat against the IC approach is dual practice (DP) legally allowed for health professionals (i.e. the combination of public and private practice) (Garattini and Padula, 2018*b*), which inevitably raises financial conflicts of interest.

The increasing plea for IC reflects the ever growing demand induced by chronic diseases of ageing and multi-morbid individuals living in community with both physical and mental needs, the major challenge of the modern European health systems. IC is certainly enhanced by a (necessarily public) single ‘employer’, being the presence of many (public and/or private) ‘players’ antithetic to IC by definition (Milstein and Blankart, 2016). The existence of several providers thwarts integration, since each actor is obviously orientated to pursue its own financial interests in the long run. According to the IC approach, also health and social services should be merged nowadays to enhance both horizontal and vertical integration (Goddard and Mason, 2017), eventually surmounting all the organisational barriers to boost continuous care.

An exclusive contract of employment for civil servants should be the sine qua non for delivering public services effectively and efficiently. In fact, any form of DP legally allowed in a health system can only mix up business and medical ethics, ultimately undermining the patients’ fiduciary relationship with health professionals (McCartney, 2018). An almost paradoxical form of DP is when public health care organisations directly provide DP in their facilities. Such an extreme form has been associated to the ethical concept of ‘institutional corruption’ (Sommersguter-Reichmann and Stepan, 2017), potentially deterring the public employers’ ability to achieve their primary goals. The ban of any form of DP is fully supported by business administration (Holmstrom, 1999). It would be very strange to allow an employee to work for two firms contemporarily, and even stranger to allow her/him to deal privately with the same clients in her/his free time, as happens for physicians with DP. At the same time, although medicine is first a mission aimed at serving people, this should not involve limitless sacrifice for health professionals, who would deserve a generous salary in a civilised society. More, once DP is forbidden, it would be much more acceptable the request of health professionals to make their employers accountable for legal expenses in case of lawsuits, so as to prevent redundant practices of defensive medicine induced only by the risk of litigation (Garattini *et al*., 2020).

## Focus on mental health care

According to the previous analysis, it is apparent that the IC approach is particularly important for long-term chronic illnesses associated with high levels of disability, such as neurodegenerative diseases and mental disorders, where the mix of health and social services needed can be very complex. In high-income countries, the combination of ageing populations and increasing prevalence of multimorbidity has been estimated around 65% in people over 65 and up to 80% for those over 85 (Català-Lopez *et al*., 2018). Therefore, nowadays the growing number of community-dwelling individuals with multiple physical and mental health needs is the major challenge for the European health systems, regardless of differences in health policies throughout single countries.

Despite growing levels of needs in many European countries, a substantial gap in access to care has been noted for some health services, such as physical rehabilitation (Garg *et al*., 2020) and mental disorders (Barbato *et al*., 2016). This happens also in public health systems, which should provide universal health coverage in principle. However, this is often limited by funding schemes biased towards acute treatments and surgical or pharmacological therapies in practice (Garattini *et al*., 2022*b*). The case of mental disorders is striking. The last findings from the Global Burden of Disease Studies show that in Western Europe the annual disability adjusted years lost (DALYs) due to mental disorders have been estimated at 28.5 million, representing 22.5% of the total disease burden (Arias *et al*., 2022; GBD 2019 Mental Disorders Collaborators, 2022). Besides these broad figures, some features of the impact of mental disorders on population health should be reminded further. People with mental health problems experience earlier mortality as much as 20 years, due to a combination of unhealthy lifestyles, iatrogenic factors, physical comorbidities, restricted access to prevention and early treatment for physical health (Nordentoft *et al*., 2013). Moreover, most mental disorders begin early in life, are long-term and increase other health care costs, especially for chronic illnesses, such as asthma, cardiovascular disorders and diabetes (Wykes *et al*., 2015).

The evolution of the burden associated to mental disorders is a further matter of concern because evidence shows an increase of its relative share in comparison with other health conditions in recent years (Rehm and Shield, 2019), pointing out the limitations and the narrow scope of the current efforts in terms of prevention and treatment. Depression represents the largest single condition contributing to the overall burden, with a prevalence in Western Europe estimated between 3 and 6% of the population, and a proportion of mental disorders DALYs around 37% (Arias de la Torre *et al*., 2021; GBD 2019 Mental Disorders Collaborators, 2022). Despite strong evidence suggesting that psychosocial treatments should be considered as the first-line interventions for depression and other common mental disorders (Furukawa *et al*., 2021), many public health systems do not guarantee users to get them and care substantially relies on drugs (Patel, 2022). This is an area in which not only the need for IC between social and health services should be a priority, but it is also necessary to target the social determinants of mental health within a broad public health perspective including aspects such as poverty, housing instability, social exclusion and social inequality (Lund *et al*., 2018). Since depression and other common mental disorders are highly prevalent, care should be offered at primary level and include psychosocial interventions and a substantial connection with social services in order to support or regain social inclusion of people with such disorders.

## Towards a common model of European health system

In the light of this theoretical exercise focused on the attempt to figure out a European health system, we are fiercely convinced that a public National Health and Social Service (NHSS) should be the most indicated model for both funding and providing health and social care in modern societies (Mur-Veeman *et al*., 2003). As to the private sector, of course it can exist in health care, like in any other domain, probably fulfilling the requests of wealthier citizens. We just argue that private and public actors can co-exist in health care, but separately. When necessary, the NHSS could recur to private providers for local catchment areas where public services are not able to cover the essential needs of resident people in due time. However, these unfulfilled needs should be estimated in advance and funded through specific budgets (not fee-for-service tariffs) to avoid undermining coordination and synergies among providers within the NHSS.

To conclude, once respected a few but clear rules of the game at the macro level, an organisational culture rooted in teamwork and collaboration at micro level should fit much better than a competitive one to manage health and social services, constraining as much as possible the unwanted effects of business and marketing in health care. The big challenge of the common European health system here envisaged would be to limit the negative influences of politics and bureaucracy. Potential remedies should be explored to limit these two major threats.
